# Integrated Rehabilitation Approach Utilizing Swiss Ball Training, Mulligan Taping, and Mobilization With Movement for Simultaneous Management of Sacroiliac Joint Dysfunction and Lateral Ankle Sprain in a Badminton Athlete: A Case Study

**DOI:** 10.7759/cureus.56942

**Published:** 2024-03-26

**Authors:** Saylee S Shedge, Swapnil U Ramteke, Subrat Samal

**Affiliations:** 1 Sports Physiotherapy, Ravi Nair Physiotherapy College, Datta Meghe Institute of Higher Education and Research, Wardha, IND; 2 Musculoskeletal Physiotherapy, Ravi Nair Physiotherapy College, Datta Meghe Institute of Higher Education and Research, Wardha, IND

**Keywords:** y balance test, sports physiotherapy, mulligan taping, movement with mobilization, carioca, sacroiliac joint dysfunction, lateral ankle sprain, badminton, core strength, swiss ball training

## Abstract

This case report details an integrated rehabilitation plan implemented for a professional badminton player who presented with issues of sacroiliac joint (SIJ) dysfunction and a lateral ankle sprain. The integrated approach aimed to address both musculoskeletal issues, considering their potential reciprocal influences on biomechanics and functional performance. The athlete underwent a thorough initial assessment, including clinical examination, imaging, and biomechanical analysis. Treatment began with targeted interventions for acute ankle sprain management, such as rest, ice, compression, and elevation (RICE) followed by progressive exercises to restore ankle stability and range of motion (ROM). Concurrently, a specialized program was devised to address the underlying sacroiliac joint dysfunction through manual therapy, therapeutic exercises, and core stabilization routines. Throughout the rehabilitation process, the focus remained on integrated exercises that targeted both the ankle and sacroiliac joint, promoting optimal neuromuscular coordination and joint function specific to badminton demands. Regular reassessments guided the progression of interventions, ensuring a personalized and athlete-centric approach. The positive outcome highlights the importance of a holistic rehabilitation strategy in managing complex musculoskeletal conditions in athletes, facilitating efficient recovery, and reducing the risk of recurrence. This case report highlights the effectiveness of an integrated approach in enhancing performance and preventing reinjury in badminton athletes facing multifaceted musculoskeletal challenges.

## Introduction

Badminton stands as one of the globally favored sports engaging nearly 200 million participants across various proficiency levels [1]. Recognized as the fastest racket sport, it involves intense shots demanding specific movements including forward lunges and backward or lateral jumps. Notably, the lower extremity is the most susceptible area, constituting 43%-86% of all injuries in the realm of badminton. The most usually injured body parts are the ankle and knee joints [2]. Numerous epidemiological research indicate that lateral ankle sprains are the most common ankle injury in badminton. Acute lateral ankle sprains are an ordinary ailment suffered by athletes participating in field, court, and indoor sports. Sprains over the lateral side of the ankle can cause a variety of motor behavioral deficits such as changes in the stiffness of the lower extremity, differences in lower extremity joint coupling, and decreased invertor and evertor strength [3]. The lack of strength in the core muscles has been associated with the occurrence of several lower limb injuries, some of which are also linked to excessive pronation of the foot [4].

The sacroiliac joint (SIJ) is a complex region encompassing bone, joint, cartilage, muscle, ligament, and nerve. Its structural integrity and correct interplay are essential for individuals to maintain their daily activities. Among its primary functions is the distribution of load originating from the trunk to the lower extremities. Consequently, dysfunction in the SIJ can cause a change in balance in the lower limb mobility affecting walking and running. In runners, SIJ pathology, characterized by conditions such as repetitive stress, articular degeneration, associated lumbar spine deformities, inflammatory processes, or complications from previous lumbar spinal fusion, can significantly disrupt the biomechanical harmony, precipitating a compensatory change in gait and mobility [5,6]. Adequate muscle activation ensures the normal transmission of loads over the lumbopelvic area [7]. The risk of lower extremity injuries appears to increase in the presence of SIJ dysfunction [8]. Athletes experiencing SIJ dysfunction or pain exhibit diverse loading patterns in the SIJ, potentially leading to varied neuromuscular activation patterns as proposed by Snijders et al. with the help of a compressive loading model [7]. The neurophysiological changes resulting from the injury can influence the pattern of muscle activation during weight transfer across the SIJ [7]. Exercise therapy is a successful approach to effectively managing recurrent ankle sprains and chronic ankle instability. Interventions such as neuromuscular training and balance exercises have demonstrated effectiveness in dealing with deficiencies in muscle strength. The strength training of weakened muscles is vital for prompt recovery and acts as a preventive measure against future injuries [9]. The typical approach to treating SIJ dysfunction through physical therapy focuses on addressing the abdomino-lumbo-sacro-pelvic-hip complex. An exercise regimen for SIJ dysfunction aims to enhance core strength and rectify muscle imbalances that could potentially amplify shearing forces on either one or both SIJs. Stabilizing the pelvis is essential for coordinated femur movement, particularly during single-leg loading movements. Pelvic girdle instability increases the risk of microtrauma injuries, especially when athletes engage in high-speed, repetitive, and asymmetrical movements during training or competitions. The center of gravity is positioned close to the sacroiliac joint, underscoring the crucial role of the SIJ in transmitting loads. Consequently, disorders affecting the SIJ can disrupt load transfer either distally to proximally or proximally to distally [10].

## Case presentation

Patient information

A 19-year-old female badminton athlete came to the sports physiotherapy outpatient department (OPD) with complaints of pain and swelling in the right ankle. The patient gives a history of twisting injury to the right ankle while playing badminton. Following the injury, there was a sudden onset of pain over the lateral aspect of the ankle around the lateral malleolus, which was progressive in nature. The patient was advised to rest for one week by the coach. After one week of rest, the athlete again resumed back to her sport. While playing, the patient experienced the same twisting injury on the right ankle. Immediately after the injury, there was a sudden onset of pain and swelling. The patient experienced extreme pain, so she visited a local hospital in Wardha where she was advised of hot water fomentation. After hot water fomentation, the patient observed an increase in swelling and pain. The patient also had difficulty in walking. So she visited another local hospital in Wardha and was advised to take an X-ray to rule out any fracture. After investigations, she was diagnosed with a right lateral ankle sprain (anterior talofibular ligament sprain). Medications were prescribed for pain relief, and she was advised to use an ankle brace for one month. The patient did not experience much pain relief even after using an ankle brace, so she was referred to a local physiotherapist in Wardha where she was treated by therapeutic ultrasound for one month. Post ultrasound therapy, she experienced very little pain relief and was then referred to sports physiotherapy OPD for further management.

Clinical findings

The patient had an ectomorphic built. On examination, the visual analog scale (VAS) score on activity was 8/10 and at rest was 3/10. Pain aggravated on ankle plantarflexion and inversion and reduced on rest. On inspection, the swelling was seen over the lateral aspect of the right ankle joint. On palpation, grade 1 pitting edema was present, and there was a presence of grade 1 tenderness around the lateral malleolus. Ankle joint range of motion was assessed using a universal goniometer, and it was found to be reduced. Muscle strength was assessed by manual muscle testing (MMT), and there was reduced strength in ankle dorsiflexors and evertors. She was advised to undergo exercise therapy for ankle instability, and taping was done for the same.

After four months, the patient visited sports OPD with complaints of low back pain over the bilateral SIJ region. The pain was gradual on onset and progressive in nature. The pain aggravated on movements such as forward bending. Due to the pain, she was not able to jump during smashing and was finding difficulty in prolonged standing. On examination, the patient reported a VAS score of 7/10 on movement and 4/10 at rest. On palpation, grade 2 tenderness was present over the bilateral SIJ. On special test examination, compression test, distraction test, and thigh thrust test reproduced symptoms and were positive bilaterally as shown in Table 1. Lumbar range of motion was assessed using a modified Schober test, and it was found to be reduced. MMT was used to assess the core muscle strength of abdominals, and trunk extensors were reduced. Based on the above assessment, the patient was diagnosed with bilateral SIJ dysfunction (posterior). A rehabilitation protocol for SIJ dysfunction was planned to reduce pain and improve core muscle strength.

**Table 1:** Diagnostic maneuvers for SIJ pain SIJ: sacroiliac joint, ASIS: anterior superior iliac spine

Test	Description
Thigh thrust (posterior shear)	Ask the patient to lie in a supine position. On the side to be tested, flex the hip to 90°. Apply posteriorly directed force through the femur and consider the test positive if it provokes pain.
Gapping (distraction)	Ask the patient to lie down in a supine position. Simultaneously position the heels of both hands on each ASIS, exerting force posteriorly and laterally. The test is positive if pain is reproduced.
Compression	Place the patient in a lateral recumbent position with the affected side up, flexing the hip to 45° and the knee to 90°. Apply a downward directed force on the anterior superior iliac crest.

Clinical investigations

**Figure 1:**
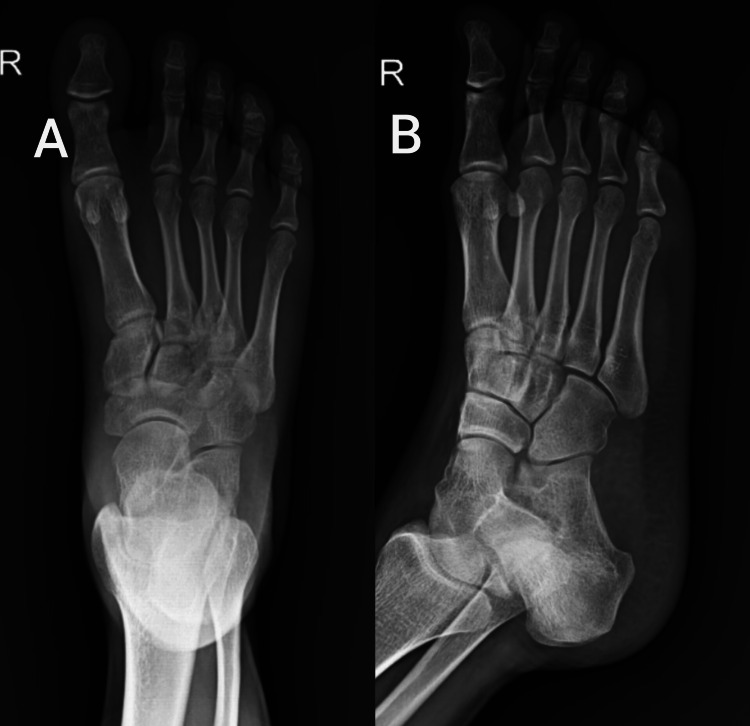
X-ray of the right ankle joint A: anteroposterior view, B: lateral view

To rule out fracture, X-ray was done, which revealed no fractures around the ankle joint. The anteroposterior view is shown in Figure 1A, and the lateral view is shown in Figure 1B.

**Figure 2:**
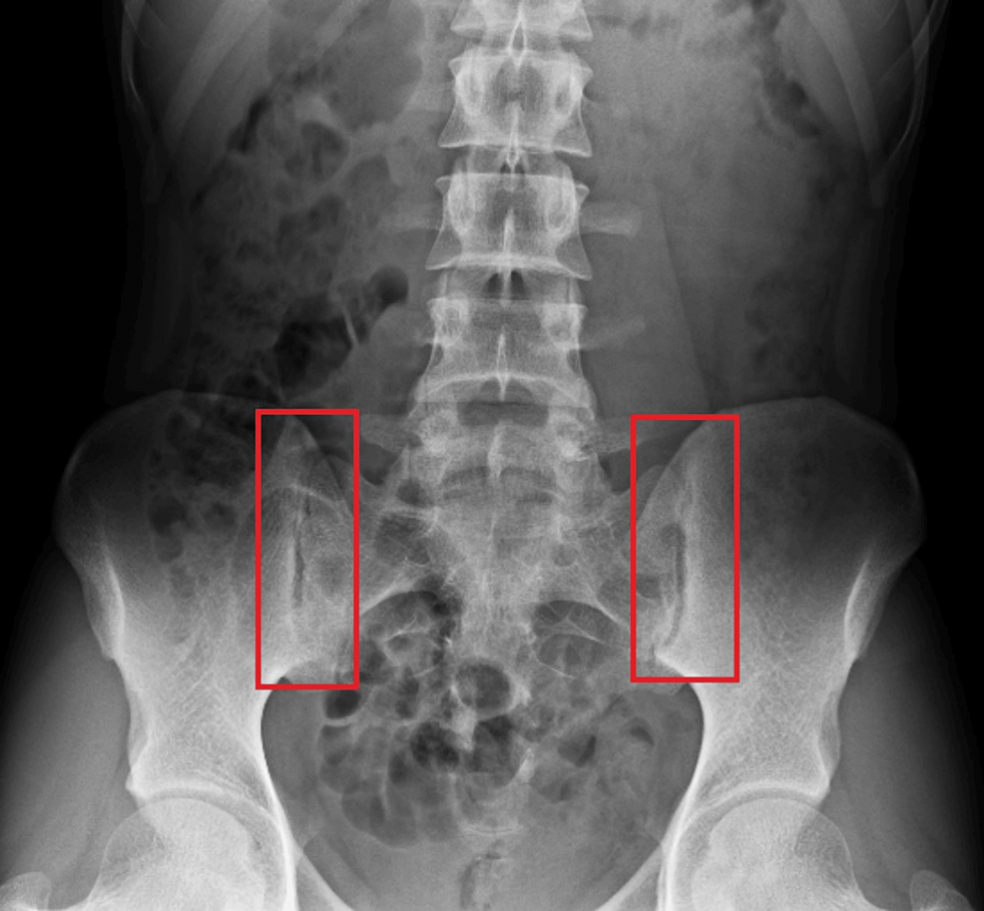
X-ray of the lumbosacral spine in the AP view showing SIJD AP: anteroposterior, SIJD: sacroiliac joint dysfunction

X-ray of the lumbosacral spine in the anteroposterior view is shown in Figure 2.

Physiotherapy intervention

Physiotherapy is considered to be an integral part in treating ankle sprains, which consists of both neuromuscular and proprioceptive exercises. Appropriate rehabilitation can prevent recurrent ankle sprains and future functional ankle instability. SIJ rehabilitation in badminton athletes is crucial for optimal performance and injury prevention. A stable SIJ ensures optimal power transfer during dynamic movements, improving smash and agility on the court. Additionally, proper rehabilitation reduces the risk of imbalance that could lead to chronic pain, promoting sustained athletic excellence. Overall, prioritizing SIJ health is integral for peak performance in badminton. In patient's care, a physiotherapy protocol for ankle sprain was framed as shown in Table 2 and for SIJ dysfunction as shown in Table 3.

**Table 2:** Rehabilitation protocol for lateral ankle sprain RICE: rest, ice, compression, and elevation, IFT: interferential therapy, TENS: transcutaneous electrical nerve stimulation, MET: muscle energy technique

Phases	Goals	Exercise/interventions
Immediate phase (day 1-7)	Reduce edema and pain	Implement RICE protocol: rest, ice (5-6 times/day for 5-10 minutes), compression (using a figure of 8 pattern with crepe bandage), and elevation; utilize IFT or TENS for pain reduction; and MET for dorsiflexors and evertors.
	Protect ligaments and soft tissues	Ankle binder to control edema, mobilization with movement (posterior fibular glide) and rigid taping, and allow weight-bearing as tolerated.
Early phase (week 2-4)	Reduce pain and swelling	Proceed with all exercises from the earlier phase.
	Promote healing	Utilize low-level laser therapy to enhance blood flow and promote healing.
	Gradually load the muscles	Towel crunch/gathering exercise; introduce dynamic concentric and eccentric exercises for invertors, evertors, plantar flexors, and dorsiflexors with manual resistance as tolerated; and calf raises in sitting and standing as tolerated.
Intermediate phase (week 4-6)	Increase muscle loading	Inversion, eversion, dorsiflexion, and plantarflexion exercises with resistance bands and incorporate hamstring and quadriceps strengthening exercises with a gradual increase in resistance.
	Start proprioception training	Initiate unilateral leg balance on a stable surface then progress to an unstable surface and introduce throwing and catching an object while balancing on one leg on a stable surface, perform unilateral leg balance while bending forward, and include balance exercises on a wobble board and trampoline.
	Progress proprioception training	Implement figure of 8 and "Z" walking and tiptoe walking for further progression.
Late phase (week 6 onward)	Return to normal activity	Proceed with all exercises from the earlier phase; initiate with jogging, integrate figure of 8 and "Z" walking with jogging; sidestepping on a stepper and carioca; progress to running; balance on wobble board and trampoline against resistance; and engage in sports-specific training under supervision.

**Table 3:** Rehabilitation protocol for sacroiliac joint dysfunction SIJ: sacroiliac joint, IFT: interferential therapy, ROM: range of motion

Goals	Exercise/intervention	Dosage
To reduce pain	IFT	80-100 Hz, 4 pole vector, 10 minutes
To improve ROM	Mulligan's mobilization with movement over the bilateral SIJ	Anterolateral glide, 10 repetitions/set for 3 sets
To strengthen core muscles	Plank, mountain climbers, and bridging	10 repetitions/set for 2 sets
To progress core muscle training	Push up on a Swiss ball, prone ball hold with mountain climber, hip extension and knee flexion, and supine lower abdominal cable curl	15 repetitions/set for 3 sets

**Figure 3:**
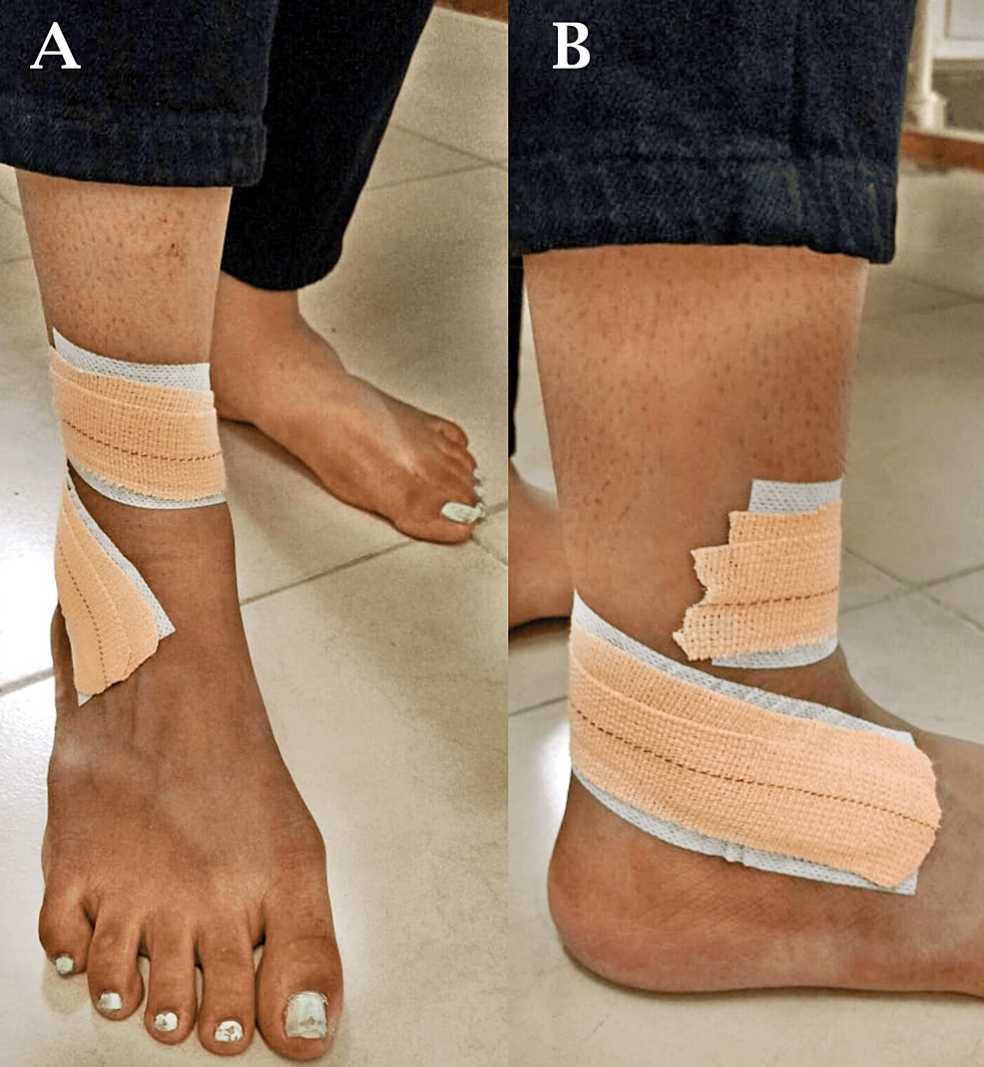
Mulligan taping for right ATFL sprain (A and B) ATFL: anterior talofibular ligament

**Figure 4:**
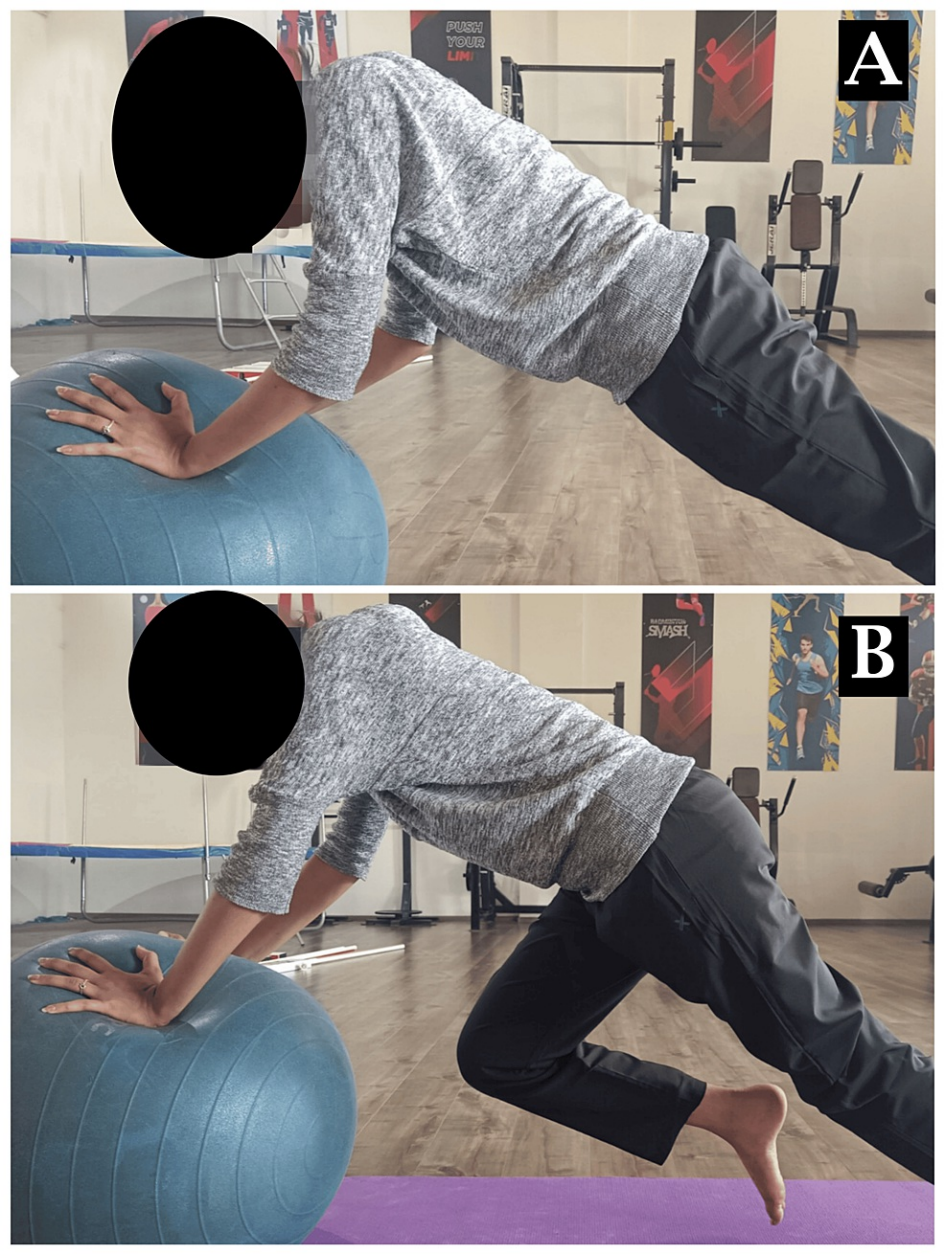
Interventions for SIJ dysfunction A: push-up on a Swiss ball, B: prone ball hold with mountain climber SIJ: sacroiliac joint

**Figure 5:**
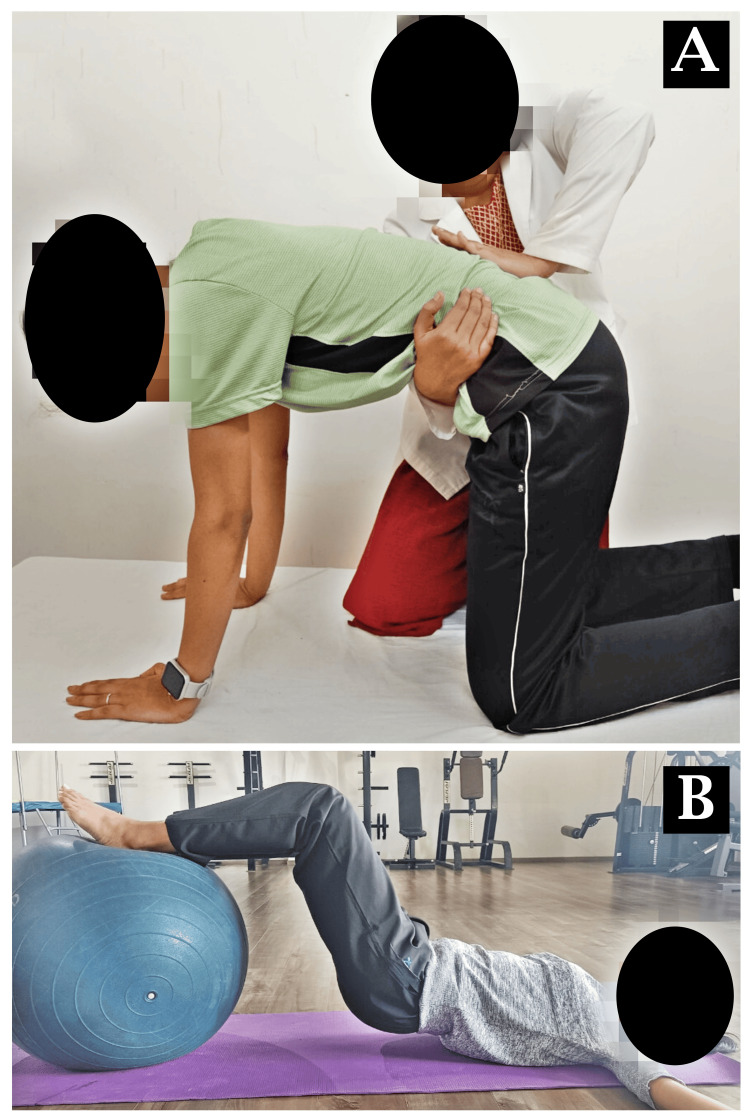
Physiotherapy rehabilitation for SIJ dysfunction A: mobilization with movement for the SIJ, B: hip extension and knee flexion SIJ: sacroiliac joint

Mulligan taping was done for right anterior talofibular ligament (ATFL) sprain as shown in Figure 3A-3B. The patient performing exercises on a Swiss ball for SIJ dysfunction is shown in Figure 4A-4B and Figure 5A-5B.

Follow-up and outcome measures

Outcome measures were assessed before and after the treatment. Ankle range of motion (ROM) was assessed using a universal goniometer, and modified Schober's test was performed by inch tape method whose findings are shown in Table 4. Muscle strength was assessed using MMT as demonstrated in Table 5. Pain was assessed using the VAS for lateral ankle sprain and SIJ dysfunction, lower quarter Y balance test was used to assess balance, and the athlete disability index questionnaire was used to find how low back pain is affecting sports and daily activities as demonstrated in Table 6.

**Table 4:** Joint range of motion assessment Normal ankle range of motion: dorsiflexion: 0°-20°, plantarflexion: 0°-55°, inversion: 0°-35°, and eversion: 0°-15°

Joint	Pre-rehabilitation	Post-rehabilitation
Ankle dorsiflexion	0°-10°	0°-15°
Ankle plantarflexion	0°-15°	0°-45°
Ankle inversion	0°-10°	0°-35°
Ankle eversion	0°-7°	0°-12°
Lumbar flexion	8 cm	5 cm
Lumbar extension	3 cm	2 cm

**Table 5:** Strength assessment (manual muscle testing) Grades of manual muscle testing for ankle: 2: full ROM with gravity eliminated, 3: full ROM against gravity, 4: full ROM against gravity, moderate resistance, 5: full ROM against gravity, maximum resistance Grades of manual muscle testing for upper abdominals: good (8): with the arms folded across the chest, the subject is able to flex the vertebral column and keep it flexed while entering the hip flexion phase and coming to a sitting position, fair (5): with the arms extended forward, the subject is able to flex the vertebral column but is unable to maintain the flexion when attempting to enter the hip flexion phase Grades of manual muscle testing for lower abdominals: good (8): with arms folded across the chest, the subject is able to keep the lower back flat while lowering the legs to an angle of 30° from the table, fair (6): with arms folded across the chest, the subject is able to keep the lower back flat on the table while lowering the legs to an angle of 60° from the table Grades of manual muscle testing for back extensors: good (4): prone with hands clasped behind the head, extends spine until trunk raised from the table but may display some signs of effort, fair (3): prone with arms at side, extends spine until the umbilicus clears the table, completes ROM ROM: range of motion

Muscles	Pre-rehabilitation	Post-rehabilitation
Ankle dorsiflexors	3/5	5/5
Ankle plantar flexors	2/5	4/5
Ankle invertors	2/5	4/5
Ankle evertors	3/5	5/5
Upper abdominals	5/10	8/10
Lower abdominals	6/10	8/10
Back extensors	3/5	4/5

**Table 6:** Outcome measures VAS: visual analog scale

Outcome measures		Pre-rehabilitation	Post-rehabilitation
VAS (right lateral ankle sprain)	On activity	8/10	2/10
	At rest	3/10	0/10
VAS (sacroiliac joint dysfunction)	On activity	7/10	1/10
	At rest	4/10	0/10
Lower quarter Y balance test		Left: 85.10%	Left: 91.15%
		Right (affected): 60.70%	Right (affected): 80.23%
Athlete disability index questionnaire		66.66%	19.88%

## Discussion

Sacroiliac joint dysfunction and lateral ankle sprains are common injuries among athletes, particularly those engaged in sports involving repetitive movements and sudden changes in direction, such as badminton. The integration of Swiss ball training, Mulligan taping, and mobilization with movement in the rehabilitation process demonstrates a holistic and innovative approach in managing these concurrent injuries. Swiss ball training offers dynamic stabilization exercises, which are crucial for enhancing core strength and stability and improving proprioception, essential components in preventing and rehabilitating sacroiliac joint dysfunction. On the other hand, Mulligan taping provides localized support and proprioceptive feedback to the ankle joint, aiding in the recovery and prevention of recurrent ankle sprains. By incorporating mobilization with movement into the rehabilitation program, the sim was to restore normal joint mechanics, improve range of motion, and alleviate pain, thereby facilitating the athlete's return to sport.

A study was conducted by García-Peñalver et al. to compare the effect of thrust and muscle energy techniques (METs) in treating SIJ dysfunction in middle-distance running athletes. The quasi-experimental design involved 60 adult athletes randomly divided into thrust, MET, and control groups. Statistically significant differences were found between the MET and thrust groups compared to the control group, with a notable reduction in positive dysfunction. The thrust technique was deemed more effective, showing positive results both immediately and in the long term, while the MET only demonstrated changes after the initial intervention [5]. Zaidi et al., in their randomized controlled trial with 60 participants, compared MET and Maitland mobilizations for SIJ dysfunction. Both groups received lumbopelvic stability exercises. After four weeks, both MET and Maitland mobilizations showed significant improvements in pain and disability with no significant difference between the two groups. The study concluded that both interventions, when combined with lumbopelvic stability exercises, are effective in treating chronic SIJ dysfunction [11].

The study by Sivakumar et al. investigated the impact of Mulligan's joint mobilization, motor control exercises, and aerobic exercises on patients with SIJ dysfunction. Mulligan's mobilization with motor control exercises demonstrated significant improvement in pain, functional ability, and transversus abdominis muscle endurance compared to motor control exercises alone. The findings suggest that combining Mulligan's mobilization with motor control exercises is an effective intervention for SIJ dysfunction [12]. Shin et al. conducted a comparative study between the efficacy of acupuncture alone and acupuncture combined with kinesiotape (AcuKT) in participants with acute lateral ankle sprains. The findings suggested that the addition of kinesiotape to acupuncture did not yield a positive add-on effect in terms of pain reduction, edema, functional recovery, daily activities, quality of life, or prevention of recurrent ankle sprains [13]. Marrón-Gómez et al., in their randomized controlled trial involving 52 participants with chronic ankle instability, stated that both mobilization with movement and talocrural manipulation (high velocity and low amplitude) significantly improved ankle dorsiflexion over 48 hours when compared to a placebo group. While both techniques demonstrated similar effectiveness, mobilization with movement showed greater effect sizes in enhancing ankle dorsiflexion [14].

The cross-sectional study by Abdollahi et al. investigated the correlation between SIJ dysfunction and pain with lower limb and pelvic girdle injuries in Iranian basketball players. Among 204 participants, those with SIJ pain demonstrated higher rates of previous pelvic girdle and lower limb injuries. Participants solely experiencing SIJ dysfunction were more likely to report acute injuries in the pelvic girdle and lower limb. The findings emphasized the significance of evaluating and addressing SIJ dysfunction/pain in the design of rehabilitation programs for sports-related injuries [7].

Overall, these findings emphasize the importance of individualized treatment approaches that combine manual therapies, exercise, and careful evaluation of underlying biomechanical factors to optimize outcomes in managing musculoskeletal dysfunctions, particularly in athletes. They also highlight the potential synergistic effects of combining diverse treatment modalities and stress the importance of tailoring rehabilitation programs to suit the specific injury patterns observed in athletes.

## Conclusions

The integration of various rehabilitation modalities is becoming increasingly prevalent in sports medicine and physical therapy, aiming to provide comprehensive care for athletes with musculoskeletal conditions. Overall, the integrated rehabilitation approach outlined in this case study underscores the importance of individualized treatment strategies used to treat SIJ dysfunction and lateral ankle sprain in a badminton athlete, showing promising results. By combining targeted exercises, manual therapy techniques, and adjunct modalities, therapists can effectively address the complex interplay of biomechanical dysfunctions and optimize the athlete's recovery process, ultimately synergizing to produce positive outcomes for the patient, thus facilitating their return to optimal function and performance in badminton, enabling a safe and successful return to sport.
